# Changes in preventive behaviour after COVID-19 vaccination in Thailand: a cross-sectional study

**DOI:** 10.1186/s12889-022-14494-x

**Published:** 2022-11-08

**Authors:** Pitchayanont Ngamchaliew, Narathip Kaewkuea, Netipong Nonthasorn, Thanawat Vonnasrichan, Natthakarn Rongsawat, Leena Rattanachai, Wannachai Chaipipattanakij, Sutthida Kamolnawin, Polathep Vichitkunakorn

**Affiliations:** 1 Department of Family and Preventive Medicine, Faculty of Medicine, Prince of Songkla University, 90110 Hat Yai, Songkhla Thailand; 2 Medical student, Faculty of Medicine, Prince of Songkla University, Hat Yai, Songkhla Thailand

**Keywords:** Protective behaviours, SARS-CoV-2, Pandemic, Public health, Preventive medicine

## Abstract

**Background:**

Protective behaviours (e.g., mask-wearing, handwashing, avoiding social gatherings) and mass vaccination are effective ways to control the coronavirus disease 2019 (COVID-19) pandemic. Previous studies found that people who get vaccinated may change their protective behaviours. The Thai government has endorsed several mix-and-match vaccine regimens to eliminate the insufficiency of each vaccine brand. This study aimed to determine levels of protective behavioural changes after COVID-19 vaccination and its relationship with various vaccine regimens in Thailand.

**Methods:**

A descriptive cross-sectional study was conducted between September 13, 2021, and January 14, 2022. Data were collected using an online questionnaire distributed via social media platforms and posters in public places in Thailand. The questionnaire comprised six items for demographic characteristics, seven items for COVID-19 vaccine regimens, and four items for protective behaviours. The vaccinated Thai population aged ≥ 18 years were surveyed. Statistical analyses included a Chi-squared test, Wilcoxon signed rank test, and multivariate logistic regressions.

**Results:**

Of the 469 participants, more than half were females (67.4%), single (57.4%), and lived in an urban area (67.2%). Significant differences were observed with regard to median scores in handwashing (5.0 vs. 5.0, *p*-value < 0.001), physical distancing (4.0 vs. 5.0, *p*-value = 0.019), and avoiding social activity (4.0 vs. 5.0, *p*-value = 0.010) in pre- and post-vaccination situations. Approximately 70–90% of the participants did not report changes in protective behaviours after vaccination. Overall, 17.4%, 13.9%, and 12.7% of participants showed improvements in avoiding social activity, physical distancing, and handwashing respectively. Multivariate analysis revealed that improvements in protective behaviours were significantly associated with the age group (between 18 and 24 years), non-healthcare worker status, and those who lived in urban areas. No significant evidence of vaccine regimens was found relative to improved protective behaviours.

**Conclusion:**

This evidence revealed that Thai people maintain their protective behaviours after vaccination but rather improved them. Moreover, demographic data were significantly associated with improved protective behaviours, but various vaccine regimens were not. These findings might be useful for implementing policies to maintain personal protective behaviours after vaccination against COVID-19.

**Supplementary Information:**

The online version contains supplementary material available at 10.1186/s12889-022-14494-x.

## Introduction

Coronavirus disease 2019 (COVID-19) is caused by the severe acute respiratory syndrome coronavirus 2 (SARS-CoV-2) virus and can lead to severe respiratory symptoms or life-threatening illnesses and even death [1] The rapid COVID-19 outbreak had a significant negative impact on the global community across all dimensions such as health, economy, society, and political systems worldwide [2–4]. To handle this outbreak, several countries implemented various preventive policies regarding protective behaviours, such as wearing masks, physical distancing, handwashing, and avoiding social gatherings, along with COVID-19 vaccination for reducing the risk of infection, morbidity, and mortality [2, 5–7]. Since the COVID-19 pandemic, many governments have implemented social distancing measures, including movement control orders and the closure of schools and other public places, in addition to restricting the number of people allowed to gather for social activities [6, 8]. Thus, a high vaccination rate and compliance with protective measures have proven to be effective ways to return to daily life and balance the competing risks of health-system collapse and economic fallout [8, 9].

Meanwhile, people who got vaccinated could change their protective behaviours against COVID-19 [10]. A study in Israel [11] found that 47.3% of vaccinated people reduced physical distancing and 21.1% reduced wearing masks in public. In a study in Bangladesh [12], abandoning sanitiser and masks (78.2%), the inclination to avoid distancing (78%), staying outside longer (74.3%), and visiting crowded places (73.8%) was found to have increased among vaccinated individuals. Therefore, despite COVID-19 vaccination, if protective behaviours are abandoned, the SARS-CoV-2 infection rate can be significantly higher and increase the spread of new strains (i.e., Omicron variant) is likely [13]. These mutations have largely occurred in the spike protein (site for antibody binding), which attributes high infectivity and transmissibility to the Omicron strain [13]. Thus, the number of patients will increase, including the hospitalization rate and mortality rate. In this way, protective behaviours are also important to control infection rates [14].

As of 2021, various types of COVID-19 vaccines have been discovered. Thailand had registered several vaccine regimens beginning with two doses of whole-pathogen inactivated vaccine (CoronaVac, Sinovac) or viral vector vaccine (AZD1222, Oxford-AstraZeneca) in February 2021 as main regimens. In the second half of 2021, many countries have already started administering booster doses in the wake of breakthrough infections, the arrival of new variants, and a decline in long-term protection [15]. Israel has been administering BNT162b2 booster (third) shots since July 2021 [16]. However, surveys conducted in several countries have shown that many people are prone to vaccine hesitancy [17, 18]. A study in Italy found that the most common reason people refused vaccination was a lack of information on the benefits and safety of vaccines [19]. Due to uncertainty in the vaccine efficacy of main regimens, the Thai government imported booster doses of messenger ribonucleic acid (mRNA) based vaccine (BNT162b2, Pfizer-BioNTech) and whole-pathogen inactivated vaccine (BBIBP-CorV, Sinopharm) in June 2021, followed by the mRNA-based vaccine (mRNA-1273, Moderna) in November 2021 [20] to eliminate concerns regarding side effects and insufficiency of vaccines. Hence, we considered the diversity of vaccine regimens as a determinant of protective behavioural changes. This study aimed to determine levels of protective behavioural changes after COVID-19 vaccination. This assessment should yield valuable information for the implementation of public policies aimed at maintaining protective behaviours even after vaccination.

## Materials and methods

### Study design and setting

This cross-sectional study was conducted among the general Thai population from September 13, 2021, to January 14, 2022. We promoted an online self-questionnaire via social media platforms (i.e., Facebook, Line application) and placed posters with a QR code for selecting participants in public places in Thailand. Each participant read the invitation message and obtained an e-consent before answering the questionnaire. Furthermore, they were informed that they could withdraw from the study at any time. We did not provide any monetary or other compensation to the participants.

### Study population and sample size calculation

**Fig. 1:**
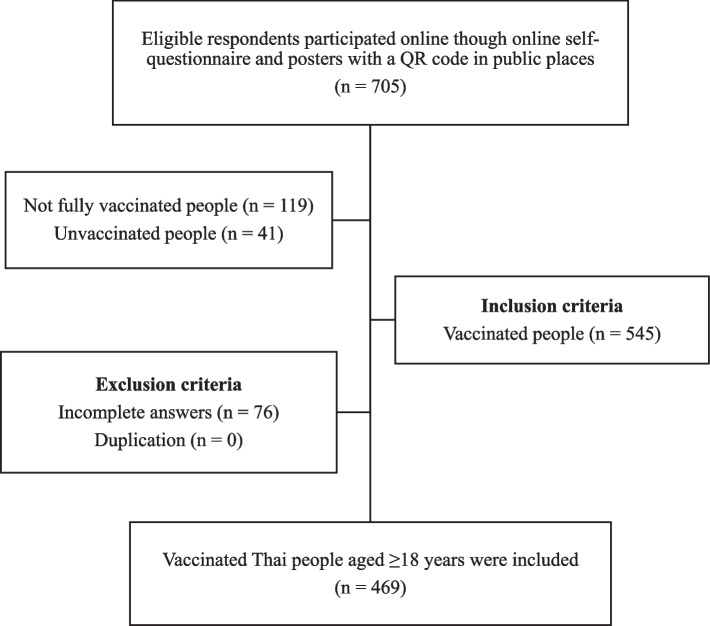
The study flowchart

The participants in our study were vaccinated Thai people aged ≥ 18 years who owned smartphones or could access Internet services. Incomplete answers were excluded. We defined the term ‘vaccinated Thai people’ as people who were fully vaccinated for more than two weeks after receiving the last dose of each regimen. For the sampling technique, we randomly recruited all participants who had completed the survey; the study flowchart is shown in Fig. 1. In calculating the sample size, the proportion of behavioural changes post-COVID-19 vaccination, (= 0.21) in Israel [11] with marginal error (= 0.04), was used to determine the target sample size for this study. Thus, a minimum of 399 participants were required for this study.

#### Study instrument

The online questionnaire was developed after reviewing published literature. It was developed based on a valid and reliable tool for the comprehensive assessment of preventive practices related to the COVID-19 pandemic (content validity ratio [CVR] = 0.81 and content validity index for scale [S-CVI/Av] = 0.97) and (Cronbach’s alpha coefficient 0.82) by Agarwal et al. [21] and DMHTT practices [distancing (D), mask-waring (M), handwashing (H), testing (T), and Thai Chana application (T)], by the Department of Disease Control of Thailand (DDC), Ministry of Public Health, Thailand [22]. The questions were validated using face validity methods by three content experts before conducting a pilot study on 30 respondents. Each item was reviewed to ensure that each item was understood similarly before testing.

The online questionnaire comprised 17 items in three sections: demographic characteristics (six items), regimens of COVID-19 vaccine (seven items), and behavioural changes post-vaccination (four items). Data were collected using an online questionnaire designed by the KoBoToolbox platform.

### Independent variables – regimens of COVID-19 vaccine and demographic characteristics

Our primary independent variable was the regimens of the COVID-19 vaccine. We arranged vaccine regimens in sequence by most regimens that the participants decided to receive (see details in Additional file 1). Demographic data consisted of information on gender, age, marital status, education, residential area, and if participants were healthcare workers (HCWs). Age was classified into four groups: between 18 and 24 years, 25 and 44 years, 45 and 59 years, and over 60 years. The residence was classified into three types: rural, suburban, and urban. Information on the HCWs variable was collected using the question, ‘Do you work in healthcare settings?’ (Answer: Yes/No).

### Dependent variable – behavioural change post-vaccination

This section was developed by using some questions (i.e., how often do you avoid going out of the house unnecessarily, how often do you wear masks while going out of home) from section A of the ‘Development and validation of a questionnaire of assessing preventive practices against COVID-19 pandemic in the general population’ (CVR = 0.81 and S-CVI/Av = 0.97) and (Cronbach’s alpha coefficient 0.82) by Agarwal et al. [21] and DMHTT practices, by the Department of Disease Control of Thailand (DDC), Ministry of Public Health, Thailand [22]. From the term DMHTT, we derived the acronym DMH which stands for distancing (D), mask-wearing (M), and handwashing (H).

This part consisted of four questions to assess the level of preventive behaviours after vaccination. The four questions comprised components of handwashing, mask-wearing, physical distancing, and social distancing (avoiding social activity). The example questions are shown in Table 1. We ranked each question on a five-point Likert scale ranging from 1 to 5 (1 = never, 2 = rarely, 3 = sometimes, 4 = often, 5 = usually). We categorized the protective behaviour change after vaccination into two groups according to behaviour frequency between pre- and post-vaccination:

**Table 1:** Questionnaire about the level of protective behavioural status after vaccination

Pre-vaccination questions	Post-vaccination questions
How often did you wear a mask when going outside one month before getting vaccinated?	How often do you wear a mask when going outside one month after being fully vaccinated for two weeks?
How often did you wash your hands after touching things in public one month before getting vaccinated?	How often do you wash your hands after touching things in public one month after being fully vaccinated for two weeks?
How often did you keep a physical distance of 1–2 m one month before getting vaccinated?	How often did you keep a physical distance of 1–2 m one month after being fully vaccinated for two weeks?
How often did you avoid social activity one month before getting vaccinated?	How often do you avoid social activity one month after being fully vaccinated for two weeks?

1. Unimproved protective behaviour: comprising two subgroups
  1.1 Unchanged level of protective behaviour: score on the pre-vaccination behaviour scale equals score on the post-vaccination behaviour scale.
  1.2 Decreased level of protective behaviour: scores on the pre-vaccination behaviour scale were at least one level higher than that of the post-vaccination behaviour.
2. Improved protective behaviour:
  2.1 Increased level of protective behaviour: score on the pre-vaccination behaviour scale was at least one level lesser than that of the post-vaccination behaviour.

### Statistical analysis

The data collected from the participants were entered into Microsoft Excel and analysed using R software version 4.1.0 [23]. Descriptive statistics (i.e., the frequency with percentage and median with interquartile range [IQR]) was used to assess the participants’ demographic characteristics and COVID-19 vaccine regimens. We checked duplicated identity across these variables including gender, age, marital status, education, healthcare worker, residence, and vaccine regimens. Demographic data and vaccine regimens were used to compare changes in protective behaviour after vaccination to enumerate their relationship. We performed a Chi-squared test for categorical variables and Wilcoxon signed-rank test for continuous variables. Multivariate logistic regressions were applied to measure the magnitude to which the independent variables affected improved protective behaviour (i.e., at least one level), with an odds ratio (OR) and a 95% confidence interval (CI). A univariate analysis was first performed to identify any potential independent variables. For multivariable analysis, potential independent variables with a *p*-value < 0.3, according to univariate analysis, were included in the initial model. A manual backward stepwise refinement was performed for the final model. That the refined model passed various assumptions (i.e., independence of observations, binary dependent variable, no influential outliers, no multicollinearity) was verified. Statistical significance was set at *p*-value < 0.05.

### Ethical considerations

The study protocol was approved by the Human Research Ethics Committee, Faculty of Medicine, Prince of Songkhla University (REC.64-365-9-1). Thorough information regarding the details and purpose of the study was provided to participants before the commencement of the survey for receiving consent. The questionnaire contained no questions on personally identifiable information.

## Results

### Participant characteristics

Among the 705 respondents, 469 (66.5%) participants were vaccinated and provided complete answers regarding behavioural change after vaccination. These 469 participants were predominantly females (67.4%), single (57.4%), and educated with a bachelor’s or higher degree (86.3%). The most prominent age group was between 25 and 44 years old (31.8%). Approximately two-thirds of the respondents lived in an urban area (67.2%) and were non-healthcare workers (HCWs) (69.4%), as shown in Table 2. There were various types of COVID-19 vaccine regimens among participants. The majority got two-dose regimens (48.7%), followed by three-dose regimens (30.2%). The most common regimens were 1st AZD1222 / 2nd AZD1222 (17.5%), followed by 1st CoronaVac/2nd CoronaVac/3rd BNT162b2 regimen (14.7%), and 1st CoronaVac / 2nd AZD1222 (14.0%), respectively.

**Table 2:** Socio-demographics characteristics and COVID-19 vaccine regimens (*n* = 469)

Socio-demographics data	n	Percentage (%)
Gender (*n* = 469)		
Female	316	67.4
Male	153	32.6
Marital status (*n* = 469)		
Single	269	57.4
Married	172	36.7
Divorced	13	2.8
Widowed	10	2.1
Other	5	1.0
Age (years) (*n* = 447)		
18–24	127	28.4
25–44	142	31.8
45–59	128	28.6
≥ 60	50	11.2
Education (*n* = 468)		
Uneducated/Primary/Elementary school	4	0.8
Secondary/High school or Vocational Certificate	60	12.8
High Vocational Certificate	30	6.4
Bachelor's Degree or higher	374	80.0
Healthcare worker (*n* = 467)		
Non-healthcare worker	324	69.4
Healthcare worker	143	30.6
Residence area (*n* = 469)		
Urban	315	67.2
Suburb	99	21.1
Rural	55	11.7
COVID-19 vaccine regimens (*n* = 469)		
Two-dose regimens		
1^st^AZD1222 / 2^nd^AZD1222	82	17.5
1^st^CoronaVac / 2^nd^AZD1222	66	14.0
1^st^BBIBP-CorV / 2^nd^BBIBP-CorV	29	6.2
1^st^BNT162b2 / 2^nd^BNT162b2	24	5.1
1^st^CoronaVac / 2^nd^CoronaVac	15	3.2
1^st^AZD1222 / 2^nd^BNT162b2	13	2.8
Three-dose regimens		
1^st^CoronaVac / 2^nd^CoronaVac / 3^rd^BNT162b2	69	14.7
1^st^CoronaVac / 2^nd^CoronaVac / 3^rd^AZD1222	38	8.1
1^st^AZD1222 / 2^nd^AZD1222 / 3^rd^BNT162b2	19	4.1
1^st^BBIBP-CorV / 2^nd^BBIBP-CorV / 3^rd^BNT162b2	15	3.2
Others		
Other regimens	99	21.1

### Protective behavioural change after COVID-19 vaccination

The characteristics of protective behaviour after vaccination included four domains: wearing masks, handwashing, physical distancing, and avoiding social activity. As shown in Table 3, there were significant differences between median scores in pre-and post-vaccination in handwashing (5.0 vs. 5.0, *p*-value < 0.001), physical distancing (4.0 vs. 5.0, *p*-value = 0.019), and avoiding social activity (4.0 vs. 5.0, *p*-value = 0.010). For wearing masks, there was no significant difference (5.0 vs. 5.0, *p*-value = 0.096).

**Table 3:** The score of protective behavioural status pre- and post-vaccination

Protective behaviour	Score from 1 to 5, median (IQR)		*P*-value
	Pre-vaccination behaviour	Post-vaccination behaviour	
Wearing mask when going outside	5.0 (5.0, 5.0)	5.0 (5.0, 5.0)	0.096
Hand washing after touching	5.0 (4.0, 5.0)	5.0 (4.0, 5.0)	< 0.001
Physical distancing of 1–2 m	4.0 (4.0, 5.0)	5.0 (4.0, 5.0)	0.019
Avoiding social activity	4.0 (4.0, 5.0)	5.0 (4.0, 5.0)	0.010

*Abbreviation*: *IQR* Interquartile range

*P*-Value: compared between score of pre- and post-vaccination behaviour, using Wilcoxon signed rank test

The individual protective behaviours after vaccination are presented in Table 4. Approximately 70–90% of the participants did not change their protective behaviours. Wearing masks was the most unchanged protective behaviour (92.1%), sequentially followed by physical distancing (82.9%), handwashing (79.5%), and avoiding social activity (71.7%). Moreover, 10.9% of the participants exhibited a decreased level of protective behaviours in avoiding social activity, followed by handwashing after touching (7.8%). In contrast, 17.4% of the participants showed improvement in avoiding social activity, followed by physical distancing (13.9%) and handwashing after touching (12.7%).

**Table 4:** Protective behavioural changes in post COVID-19 vaccination classified by demographic data and vaccine regimens (*n* = 469)

Factor	Wearing mask when go outside (%)				Hand washing after touching (%)				Physical distancing 1–2 m (%)				Avoiding social activity (%)			
	Unimproved		Improved	*P*-value	Unimproved		Improved	*P*-value	Unimproved		Improved	*P*-value	Unimproved		Improved	*P*-value
	Unchanged	Decrease	Increase		Unchanged	Decrease	Increase		Unchanged	Decrease	Increase		Unchanged	Decrease	Increase	
**Total (** ***n*** ** = 469)**	92.1	5.5	2.4		79.5	7.8	12.7		82.9	3.2	13.9		71.7	10.9	17.4	
**Gender**																
Male (*n* = 153)	84.3	10.5	3.3	0.567	80.4	6.5	13.1	1.000	81.0	3.9	14.4	0.909	64.7	11.8	22.9	0.036*
Female (*n* = 316)	92.1	2.8	1.9		77.2	8.2	12.3		81.6	2.8	13.3		72.8	10.1	14.2	
**Status**																
Single (*n* = 269)	88.5	5.2	3.0	0.485	76.2	8.9	13.4	0.850	80.7	2.2	14.9	0.252	68.0	13.4	16.7	0.257
Married (*n* = 172)	90.7	5.8	1.7		80.8	5.8	11.6		80.8	5.2	13.4		72.1	5.8	19.2	
Others (Divorced, Widowed, Others) (*n* = 28)	92.9	3.6	0.0		82.1	7.1	10.7		92.9	0.0	3.6		78.6	14.3	7.1	
**Age group**																
Early adult 18–24 (*n* = 127)	85.0	7.1	3.1	0.361	68.5	13.4	15.7	0.234	74.8	3.9	18.1	0.295	64.6	15.0	18.1	0.191
Middle adult 25–44 (*n* = 142)	93.0	2.8	1.4		83.1	7.0	8.5		83.1	0.7	14.8		72.5	12.7	13.4	
Late adult 45–59 (*n* = 128)	90.6	4.7	3.1		78.9	5.5	15.6		87.5	2.3	10.2		77.3	5.5	16.4	
Elderly ≥ 60 (*n* = 50)	88.0	8.0	0.0		80.0	4.0	12.0		76.0	6.0	14.0		60.0	10.0	26.0	
**Education**																
Vocational Certificate or below (*n* = 64)	84.4	6.3	3.1	0.964	71.9	12.5	12.5	1.000	75.0	3.1	18.8	0.257	68.8	6.3	20.3	0.504
High Vocational Certificate or higher (*n* = 404)	90.3	5.2	2.2		79.2	6.9	12.6		82.4	3.2	12.9		70.3	11.4	16.6	
**Healthcare worker**																
Non-healthcare worker (*n* = 324)	88.9	5.6	2.8	0.566	80.2	6.2	11.7	0.490	79.0	3.4	15.7	0.072	69.4	8.3	19.8	0.029*
Healthcare worker (*n* = 143)	90.9	4.9	1.4		73.4	11.2	14.7		86.7	2.8	9.1		71.3	16.1	11.2	
**Accommodation**																
Urban (*n* = 315)	88.9	5.1	2.9	0.540	77.1	8.3	12.4	0.907	81.6	3.2	13.3	0.651	67.3	11.7	18.7	0.110
Suburb (*n* = 99)	92.9	4.0	1.0		80.8	7.1	12.1		80.8	2.0	16.2		71.7	9.1	17.2	
Rural (*n* = 55)	87.3	9.1	1.8		80.0	5.5	14.5		81.8	5.5	10.9		83.6	7.3	7.3	
**Vaccine regimens**																
** 2 doses**				0.945				0.839				0.348				0.742
1^st^AZD1222 / 2^nd^AZD1222 (*n* = 82)	91.3	6.3	2.5		86.3	3.8	10.0		80.0	5.0	15.0		66.7	12.3	21.0	
1^st^CoronaVac / 2^nd^AZD1222 (*n* = 66)	93.7	4.8	1.6		80.0	3.1	16.9		87.7	1.5	10.8		78.1	4.7	17.2	
1^st^BBIBP-CorV / 2^nd^BBIBP-CorV (*n* = 29)	93.3	3.3	3.3		82.8	6.9	10.3		79.3	6.9	13.8		86.2	6.9	6.9	
1^st^BNT162b2 / 2^nd^BNT162b2 (*n* = 24)	83.3	8.3	8.3		69.6	17.4	13.0		77.3	4.5	18.2		59.1	18.2	22.7	
1^st^CoronaVac / 2^nd^CoronaVac (*n* = 15)	100.0	0.0	0.0		73.3	6.7	20.0		73.3	0.0	26.7		66.7	13.3	20.0	
1^st^AZD1222 / 2^nd^BNT162b2 *(n* = 13)	73.3	13.3	13.3		84.6	7.7	7.7		92.3	0.0	7.7		84.6	7.7	7.7	
** 3 doses**																
1^st^CoronaVac / 2^nd^CoronaVac / 3^rd^BNT162b2 (*n* = 69)	88.1	6.0	6.0		74.6	10.4	14.9		79.1	1.5	19.4		74.6	10.4	14.9	
1^st^CoronaVac / 2^nd^CoronaVac / 3^rd^AZD1222 (*n* = 38)	94.7	2.6	2.6		76.3	5.3	18.4		92.1	2.6	5.3		78.9	5.3	15.8	
1^st^AZD1222 / 2^nd^AZD1222 / 3^rd^BNT162b2 (*n* = 19)	100.0	0.0	0.0		84.2	10.5	5.3		68.4	5.3	26.3		84.2	5.3	10.5	
1^st^BBIBP-CorV / 2^nd^BBIBP-CorV / 3^rd^BNT162b2 (*n* = 15)	100.0	0.0	0.0		85.7	0.0	14.3		85.7	0.0	14.3		57.1	14.3	28.6	
** Others**																
Others (*n* = 99)	90.7	7.2	2.1		77.8	12.1	10.1		85.9	4.0	10.1		63.9	16.5	19.6	

*P*-Value: compared between improved and unimproved group, using Chi-square test

Unchanged (pre-vaccination behaviour scale = post-vaccination behaviour scale)

Decrease (pre-vaccination behaviour scale > post-vaccination behaviour scale)

Improve (pre-vaccination behaviour scale < post-vaccination behaviour scale)

According to univariate analysis, there were statistically significant differences by demographic characteristics such as gender (*p*-value = 0.036) and HCWs status (*p*-value = 0.029) in avoiding social activities. Interestingly, the analysis considered those with two-dose, three-dose, or the whole picture of vaccine regimens, but no evidence of vaccine regimens was found to be significant in relation to improved protective behaviours.

### Predicting factors of improved protective behaviours after COVID-19 vaccination

Multiple logistic regression showed that the age group between 18 and 24 years demonstrated significantly higher improvement in physical distancing (adjusted OR [AOR] 3.93; 95% CI 1.26–12.27) compared to the age group between 25 and 44 years. Non-HCWs showed significantly higher improved handwashing (AOR 4.31; 95% CI 1.72–10.94) and avoiding social activities (AOR 2.45; 95% CI 1.13–5.33). Besides, those living in urban areas showed greater improvement in avoiding social activities than those living in rural areas (AOR 3.60; 95% CI 1.11–11.64), as shown in Table 5.

**Table 5:** Logistic regression model predicting improved protective behaviours after COVID-19 vaccination (*n* = 469)

Factor	Adjusted OR (95% CI)			
	**Wearing mask when go outside**	**Hand washing after touching**	**Physical distancing 1–2 m**	**Avoiding social activity**
**Gender (ref. = female)**				
Male	2.13 (0.54–8.44)	1.17 (0.63–2.19)	0.89 (0.47–1.68)	1.58 (0.90–2.76)
**Marital status (ref. = single)**				
Married	0.71 (0.11–4.61)	1.44 (0.63–3.31)	1.00 (0.45–2.25)	1.12 (0.53–2.37)
Other	< 0.01 (0.00-inf)	< 0.01 (0.00-inf)	0.89 (0.20–3.39)	2.81 (0.05–1.44)
**Age group (ref. = middle adult, 25–44 years)**				
Early adult (18–24 years)	2.43 (0.22–27.48)	1.29 (0.45–3.74)	3.93 (1.26–12.27)*	2.36 (0.84–6.60)
Late adult (45–59 years)	2.53 (0.37–17.18)	0.59 (0.26–1.38)	1.99 (0.86–4.65)	1.33 (0.61–2.90)
Elderly (≥ 60 years)	< 0.01 (0-inf)	4.09 (0.10–1.62)	2.17 (0.58–8.09)	2.9 (0.97–8.67)
**Education (ref. = high vocational certificate or higher)**				
Vocational certificate or lower	2.71 (0.36–20.64)	1.57 (0.66–3.88)	0.81 (0.29–2.23)	2.10 (0.90,4.91)
**Healthcare worker (ref. = healthcare worker)**				
Non-healthcare worker	2.81 (0.45–17.54)	4.31 (1.72–10.94)*	0.88 (0.41–1.90)	2.45 (1.13–5.33)*
**Accommodation (ref. = rural)**				
Urban	4.29 (0.32–57.21)	1.42 (0.49–4.12)	0.89 (0.34–2.32	3.60 (1.11–11.64)*
Suburb	0.72 (0.03–16.37)	1.52 (0.48–4.84)	1.19 (0.41–3.50)	2.63 (0.75–0.92)
**Vaccine regimens (ref. = 1** ^**st**^ ** AZD1222 / 2** ^**nd**^ ** AZD1222)**				
** 2 doses**				
1^st^CoronaVac / 2^nd^AZD1222	0.3 (0.01–6.32)	0.67 (0.22–2.13)	3.07 (0.98–9.58)	0.99 (0.49–3.75)
1^st^BBIBP-CorV / 2^nd^BBIBP-CorV	< 0.01 (0.00-inf)	1.19 (0.29–4.67)	1.39 (0.30–6.53)	0.50 (0.09–2.76)
1^st^BNT162b2 / 2^nd^BNT162b2	2.69 (0.13–56.65)	1.45 (0.29–7.12)	2.30 (0.46–11.41)	1.30 (0.27–6.22)
1^st^CoronaVac / 2^nd^CoronaVac	3.1 (0.22–44.62)	2.71 (0.68,11.73)	2.63 (0.54–12.80)	1.81 (0.41–8.01)
1^st^AZD1222 / 2^nd^BNT162b2	< 0.01 (0.00-inf)	0.9 (0.09–8.43)	0.85 (0.09–8.20)	0.57 (0.06–5.31)
** 3 doses**				
1^st^CoronaVac / 2^nd^CoronaVac / 3^rd^BNT162b2	1.3 (0.14–11.99)	2.61 (0.88–7.63)	1.42 (0.42–4.78)	1.50 (0.52–4.31)
1^st^CoronaVac / 2^nd^CoronaVac / 3^rd^AZD1222	0.73 (0.06–9.60)	0.48 (0.10–2.48)	1.85 (0.54–6.40)	0.99 (0.30–3.27)
1^st^AZD1222 / 2^nd^AZD1222 / 3^rd^BNT162b2	1.08 (0.46–256.53)	2.02 (0.42–10.02)	0.57 (0.06–5.28)	0.21 (0.02–1.91)
1^st^BBIBP-CorV / 2^nd^BBIBP-CorV / 3^rd^BNT162b2	< 0.01 (0.00-inf)	0.93 (0.15–5.76)	0.86 (0.09–8.48)	0.29 (0.65–12.69)
**Other regimens**				
Others	1.09 (0.13–8.96)	0.84 (0.30–2.31)	0.82 (0.27–2.52)	1.67 (0.71–3.95)

*Abbreviation*: *OR* Odds ratio, *CI* Confidence interval

* *p*-value < 0.05

## Discussion

### Behavioural change in post-COVID-19 vaccination

Regarding the behavioural changes post-COVID-19 vaccination, this study revealed that almost vaccinated participants continued engaging in protective behaviours. Wearing masks was the most unchanged protective behaviour (92.1%), followed by physical distancing (82.9%) and handwashing (79.5%). Small groups of people exhibited an improved trend in exercising protective behaviours, especially avoiding social activity (17.4%) followed by physical distancing (13.9%) and handwashing (12.7%). Our findings were inconsistent with similar evidence found globally [10, 12, 24–26]. A possible explanation is that mixed and combined COVID-19 vaccine regimens were generally used in Thailand. For instance, two doses of whole-pathogen inactivated vaccine (CoronaVac or BBIBP-CorV) or viral vector vaccine (AZD1222), followed by booster doses of mRNA-based vaccines (BNT162b2 or mRNA-1273). That there might be uncertainty regarding vaccine novelty, unapproved efficacy of mixed regimens, and concerns regarding side effects of various mix-and-match vaccine regimens, may affect the maintenance of protective behaviours among vaccinated Thai people.

Our study results support the evidence from a previous study conducted in China [24] in March 2021, which revealed that the rate of mask-wearing did not reduce significantly after vaccination. In addition, another study from China showed that the scores of protective behaviours in the post-vaccination group were statistically higher than those in the pre-vaccination group [25]. Moreover, a previous study by Wright et al. reported that there was no clear evidence that compliance with social distancing had reduced among vaccinated UK adults relative to those who were not yet vaccinated [26]. However, our results were inconsistent with the results of the study conducted in Israel [10] between 25 March and 7 April 2021, which reported decreased physical distancing and mask-wearing in specific populations following vaccination. Additionally, a study conducted in Bangladesh [12] between 28 July and 13 August 2021 reported an increase in avoiding social distancing, handshakes, abandonment of sanitiser and masks, visits to crowded places, travelling, and staying outside for longer periods among vaccinated individuals. These results varied across countries depending on their respective public health policies. In Thailand, the government policies regarding wearing masks, public gatherings, and delaying the reopening or limiting the opening time of entertainment places were strict [27]. Likewise, in China, government policies regarding wearing masks in public places aimed at ‘zero-COVID’ were very strictly implemented [28]. Meanwhile, in other countries, especially Israel and the UK, the restrictions on public gatherings and wearing masks outdoors were lifted [29]. Government policies have to be stringent to make people adhere to protective behaviours to balance the risks of health-system collapse and economic fallout.

Moreover, at the time of data collection for Israel and the UK studies, there was evidence regarding the BNT162b2 and AZD1222 vaccine’s efficacy to protect people from SARS-CoV-2 infection [29, 30]. However, now, as new variants have arisen due to mutations of SARS-CoV-2 (i.e., Omicron variant), which have the attributes of high infectivity and transmissibility, current COVID-19 vaccine regimens might not be able to cover all strains of SARS-CoV-2 infection [13]. Therefore, at the time of data collection in our study, the Thai population was concerned about the efficacy of mixed regimens to protect them from SARS-CoV-2 infection and thus were still strictly adhering to personal protective behaviours.

### Predicting factors of improved protective behaviours after COVID-19 vaccination

Regarding predicting factors of improved protective behaviours, the overall direction of the results indicated that non-HCWs, those living in urban areas, and people aged between 18 and 24 years were significant independent predictors of improvements in handwashing, physical distancing, and avoiding social activities. Interestingly, the variety of COVID-19 vaccine regimens was not significantly related to improvements in protective behaviours after vaccination.

According to our analysis, non-HCWs exhibited significant improvements in handwashing (4.31 folds) and avoiding social activities (2.45 folds) after vaccination when compared with HCWs. Vaccinated Thai people received COVID-19 preventive recommendations after vaccination, which might have led to improved knowledge and protective behaviours among non-HCWs. Fujii et al. [31] suggested that higher perceived effectiveness might be a common factor to encourage the practice of protective behaviours in response to the COVID-19 pandemic. The relevance of this assumption is in line with a previous study, where Fujii R et al. [31] reported that people who changed their behaviours because of recommendations from doctors or public health officials were more likely to engage in handwashing/using hand sanitisers in China, Italy, and Korea. Hence, non-HCWs improved in handwashing, while HCWs already had this knowledge and usually washed their hands as a routine hygiene practice. Moreover, the people who improved their behaviours because of recommendations from doctors or public health officials were more likely to engage in avoiding social gatherings in the USA. Importantly, this study revealed that after vaccination, approximately 90% of HCWs maintain their protective behaviours, including physical distancing (95.8%), wearing masks (92.3%), and handwashing (88.1%), except avoiding social activity (82.5%). A possible explanation can be that during the COVID-19 situation, HCWs were on the frontline against COVID-19 and were unable to avoid social activities such as meetings and teamwork. These results are inconsistent with those of a study conducted by Rahamim-Cohen et al. [10] in Israel, where HCWs exhibited a minimal decrease in mask-wearing (4.3%) but a more widespread decrease in social distancing (43.5%).

Besides this, improvements in avoiding social activities were 3.6 folds higher among people living in urban areas compared to those in rural areas. A possible explanation might be that the enforcement of the Thai government’s policies such as lockdowns and night-time curfews were stricter for urban areas compared to rural areas. In urban areas, the opening and closing of services had a set time and restrictions were imposed by government officials [27]. This result is inconsistent with a previous study, where Fujii R et al. [31] reported that those who lived in urban areas were more likely to avoid social gatherings in Korea, but an inverse association was found in Italy and the UK.

Notably, our findings show that physical distancing was 3.93 folds higher among early adults than among middle adults. It might be because early adults were at a studying age when the government enforced a strategy of closing onsite educational institutions across the country and replacing them with online classrooms. Thus, early adults could practice physical distancing, before returning to onsite classrooms during the data collection for this study. This result was inconsistent with the Rahamim-Cohen study, which reported that people under the age of 50 were more likely to decrease social distancing (56.1%) as compared to those over the age of 50 (41.8%) [10]. The results can be seen against the backdrop that higher age was a known risk factor for a more severe COVID-19-associated illness and death than middle and young age [32–34]. Thus, the higher age group could be more likely to engage in protective behaviour even after vaccination than the lower age group. Therefore, the government should promote COVID-19 information and public policies among people, especially those belonging to higher age groups, to maintain protective behaviours after vaccination and prevent severe illness and death. Regarding other protective behaviours, the study at Southern U.S. university revealed that 31.4% of college students did not practice frequent handwashing during the COVID-19 pandemic compared to 33.1–61.5% reported in the pre-COVID-19 era; handwashing in early adults remains a matter of concern in these times [35, 36].

## Strengths and limitations

This study is one of the few studies that assess the levels of protective behaviours in the light of various COVID-19 vaccine regimens which were available in Thailand. This study makes a significant contribution to the literature because, as opposed to the evidence emerging from other countries regarding the relaxation of protective behaviours, these results indicate compliance with protective behaviours after vaccination. It is plausible that a few limitations may have influenced the results obtained. First, due to the situation of COVID-19, we collected data using self-reported online questionnaires. The online questionnaire may have been inaccessible to those without the internet. Moreover, there are issues with the reliability of responses in self-reported questionnaires. Second, the questions about protective behaviours sought a response on a 5-point Likert scale. It means that one level change in behavioural scale might not be significant for interpretation. Additionally, the term frequency among each individual might be different. There could be information bias, and observational studies or behavioural monitoring tools are a better way to reduce the same. Third, the questions on vaccine regimens were self-reported, and the possibility of invalid answers or recall bias should be considered. Fourth, due to the cross-sectional design, pre- and post-vaccination questions were asked at the same time; therefore, recall bias should be of concern. Lastly, in long-term data collection, variations in available types of COVID-19 vaccine, morbidity, and mortality rate by situation and time can affect periodic protective behaviours.

## Implications and further studies

Our study findings shed some light on the levels of protective behaviours pre-and post-COVID-19 vaccination and predictive factors associated with improved protective behaviours.

This assessment would yield valuable information for the implementation of public policies aimed at maintaining protective behaviours after vaccination. Furthermore, the findings of this study will help in designing targeted interventions to promote protective behaviours. Further studies should obtain a large sample, distribute online questionnaires more broadly via a cooperative institution, and advertise more in diverse work fields. The data collected on vaccine regimens from vaccine certificates or passports may help avoid invalid answers or recall bias. This study was conducted over a long period with uncertain fluctuating circumstances in Thailand; thus, data collection during a brief period with a greater quantity of responses would minimize information bias to affect behavioural change.

## Conclusion

The study results revealed that almost all Thai participants extended their protective behaviours after being vaccinated. Demographic data (i.e., early adults, non-HCWs, and those who lived in urban areas) were significantly associated with improved protective behaviours, but various COVID-19 vaccine regimens were not. These findings can be useful in the implementation of public policies, particularly in improving facial covering among the population and reducing social gatherings, and in ensuring personal protective behaviour is maintained after vaccination against COVID-19.

## Supplementary Information

**Additional file 1.** 

## Data Availability

The datasets used and/or analysed during the current study are available from the corresponding author upon reasonable request.

## Abbreviations

COVID-19: Coronavirus disease 2019
SARS-CoV-2: Severe acute respiratory syndrome coronavirus 2
HCW: Healthcare workers
CVR: Content validity ratio
S-CVI/Av: Content validity index for scale
DDC: Department of Disease Control of Thailand
IQR: Interquartile range
