# Supplementary material for: Changes in preventive behaviour after COVID-19 vaccination in Thailand: a cross-sectional study
Source: BMC Public Health. 2022 Nov 8;22:2039. doi: 10.1186/s12889-022-14494-x (PMC9640894; doi:10.1186/s12889-022-14494-x)
Supplement: Supplementary file 1 — Additional file 1. [file 12889_2022_14494_MOESM1_ESM.docx]

**Appendix 1.** Regimens of COVID-19 vaccine available in Thailand.

1. 1^st^AZD1222 / 2^nd^AZD1222 regimen
2. 1^st^CoronaVac / 2^nd^AZD1222 regimen
3. 1^st^BBIBP-CorV / 2^nd^BBIBP-CorV regimen
4. 1^st^BNT162b2 / 2^nd^BNT162b2 regimen
5. 1^st^CoronaVac / 2^nd^CoronaVac regimen
6. 1^st^AZD1222 / 2^nd^BNT162b27 regimen
7. 1^st^CoronaVac / 2^nd^CoronaVac / 3^rd^BNT162b2 regimen
8. 1^st^CoronaVac / 2^nd^CoronaVac / 3^rd^AZD1222 regimen
9. 1^st^AZD1222 / 2^nd^AZD1222 / 3^rd^BNT162b2 regimen
10. 1^st^BBIBP-CorV / 2^nd^BBIBP-CorV / 3^rd^BNT162b211 regimen
11. Other regimens
